# Asymmetry in snow-water nexus in mountain areas mainly governed by meteorological seasonal changes

**DOI:** 10.1038/s41598-025-29597-9

**Published:** 2025-11-25

**Authors:** R. Pimentel, C. Notarnicola

**Affiliations:** 1 Fluvial Dynamics and Hydrology, Andalusian Institute for Earth System Research, University of Cordoba, Córdoba, Spain; 2 Department of Agronomy, Unit of Excellence María de Maeztu (DAUCO), University of Córdoba, Córdoba, Spain; 3 Institute for Earth Observation, EURAC Research, Bolzano, Italy

**Keywords:** Streamflow, Snow cover changes, Mountain areas, Satellite images, Meteorological variables, Snow-water nexus, Hydrology, Cryospheric science

## Abstract

Despite the importance of snow cover and related streamflow changes in mountain catchments worldwide, hitherto the quantification of their mutual variability has not been addressed at this scale. This work utilizes satellite and ground observations spanning the last two decades to investigate the nexus between snow cover and streamflow across 548 mountain catchments globally. The results show that around 5% of these catchments are simultaneously affected by significant trends in both variables. This happened mainly in the Andes Cordillera, where snow season ends 28 days earlier, with a snow cover area reduction of about 15%, and a decrease in annual streamflow of 67 m^3^/s. Moreover, in 16% of the catchments, significant changes occur only in one of the variables. This asymmetric behaviour was connected with significant changes in a specific season. When the most affected season is summer, with marked temperature increase, snow changes are not reflected in streamflow, as the latter mainly depends on spring snowmelt, as observed in the European Alps. When there is an increase in winter solid precipitation, this may not be reflected in yearly snow cover changes but can impact the resulting streamflow, as shown in the western North American catchments.

## Introduction

Mountain areas are particularly prone to the effects of climate warming. Indeed, their orographic characteristics and land heterogeneity can create conditions that may exacerbate climate changes^1^. Among the key elements to be monitored, seasonal snow cover has raised a lot of attention, specifically because, being mountains the water tower for the downstream areas, changes of snow presence and duration severely impact the water availability with consequences on the environment and human water demand^2^.

When focusing on the last decades, taking advantage of a wide distribution and availability of in-situ measurements, satellite observations and modelling simulations, several studies pointed out that snow cover area (SCA) and other parameters, e.g. snow water equivalent (SWE) and/or snow cover duration (SCD), have undergone a decline in global mountain regions, even though most of the studies reported a high variability at regional scale^3–5^. In Beniston et al.^6^, many European mountain ranges in Spain, Romania, Croatia and Bulgaria reported a snowpack decline. The European Alps present a negative trend, especially below 2000 m. More specifically, Matiu et al.^7^ exploiting a large data set of ground measurements over European Alps found that over all stations and all months, 87% of the trends were negative and 13% positive, with marked changes in the spring months and at lower elevations. Similar declining trends are found in High Mountain Asia^8^, in North and South America^9,10^ even though always accompanied by a high variability. By analysing a huge dataset of satellite images, in the period 2000–2018, it was found that around 78% of global mountain areas with significant changes are undergoing a snow decline characterized by snow cover duration decreasing up to 43 days, and a snow cover area decreasing up to 13%^11^. These trends are further confirmed on a longer time series from 1982 to 2020 with an overall negative trend of − 3.6% ± 2.7% for yearly SCA and of − 15.1 days ± 11.6 days for snow cover duration^12^. While persistent snow (snow lasting around six months) showed a decline of 7.79% in the last 44 years^13^. For snow-cover duration and persistence, there is a significant decrease particularly at higher latitudes due to earlier spring melt and, in some cases, later autumn onset of snow cover^11,14–16^.

The fate of mountain snow cover follows the trends found at continental scale. For the Northern Hemisphere, Loujus et al.^17^ reported a snow mass base decreased by 46 Gt/decade across North America while the trend was negligible across Eurasia: both continents exhibit high regional variability. By using a model ensemble, Mudryk et al.^18^ reported similar results with a snow mass decrease of 5Gt/y in the period from 1981 to 2018 over the Northern Hemisphere. Gottlieb & Mankin^4^ exploited both remote sensing data and modelling to show how the snow loss on the Northern Hemisphere can be mainly attributed to human influence. Through modelling simulations, indeed, it was shown that under only internal climate variability, the actual snow loss would not have been possible.

As stated before, snowmelt water from mountain catchments is a non-negligible part of the streamflow volume, being snowmelt one of the hydrological components more affected by the actual climate change context^19^. Nevertheless, when analysing streamflow trends and their relationship with snow changes in mountain catchments worldwide, the patterns found are not homogeneous^20–23^. In Asian mountains, a non-clear streamflow trend was found when analysing the Hindukush Karakoram Himalaya region as a whole; however, for some specific catchments, positive and negative trends were found^24^. Also in Asia, in the southern face of the Tianshan mountains, Shen et al.^25^ observed a significant increase in streamflow during winter and spring for the period 1961–2010. The same pattern was observed on the northern face of the same range, but in this case for the autumn and spring months^26^. In the case of North America, snow-driven catchments in the United States showed a widespread trend towards a mean annual streamflow decline^27^, with an earlier snowmelt-streamflow volume caused by changes in the combination of precipitation and temperature patterns for the Western catchments^28^ and only by temperature in the Eastern ones^29^. In South America, for Andean catchments, different behaviour has been reported along the mountain range. An overall negative trend has been found since the early 20th in the central part of Central Andes and in northern Patagonia. However, this did not happen in other catchments of the Cordillera such as the northern part of the central Andes or central Patagonia. In these catchments, a fluctuation around the long-term mean results in drier and wetter conditions^30^. In Europe, when having a look at the European Alps, the annual median streamflow does not show a clear trend over the whole mountain range, but some significant trends are found when analysing catchments individually. For instance, a positive trend in streamflow is found in catchments driven by glaciers^31^. Similar patterns are found in the Pyrenees, where a recent study shows that most of the streamflow series analysed do not present statistically significant trends when the complete range was analysed, but for specific catchments located in the western part on the mountain range a positive significant trend was found due to a change of snowfall into precipitation^32^. Therefore, one key and challenging question regards how and under which circumstances these snow cover changes are linked and impact the downstream streamflow.

Within this context, this work aims to evaluate the nexus between snow and streamflow in mountain areas worldwide in the last two decades, by addressing the following research questions: Do significant changes in SCA determine significant changes in streamflow? Are there cases where SCA changes do not reflect streamflow variability? Where is streamflow variability not related to SCA changes? To answer them, the study exploits observations of snow and streamflow that are openly available. We selected as main variables Snow Cover Area (SCA) and snow phenology in terms of Snow Cover Duration (SCD), First Snow Day (FSD), Last Snow Day (LSD), and mean of streamflow (Qmean) to characterize the snow and hydrological conditions of the catchments, respectively. These are variables that can be easily derived from satellite and in-situ observations. The study area covers the global mountain ranges where several catchments were selected based on data availability. In the paper, we evaluate the relationship between the two variables, SCA and Qmean, addressing the mutual influence at the catchment level in global mountain regions. Moreover, as SCA only represents surface conditions, we investigated the SWE values derived from reanalysis and the relationship between SCA, SWE and streamflow. The innovative aspects of the proposed work are the following:

- To exploit for the first time snow phenology at global scale in connection with streamflow to investigate their mutual relationship.
- To generate a global view of the trends in snow and water impact in the last two decades.
- To identify patterns of changes in relation to meteorological drivers such as temperature and precipitation (both liquid and solid).

It is important to note that analyzing data from the 23-year period (2000–2022) may not be sufficient to attribute long-term climatological trends. The focus of this study is on examining intra-annual variability in snow and associated streamflow. Therefore, any reference to ‘trend’ in the following text pertains strictly to statistical trends and should not be interpreted as climatological in nature. Moreover, notwithstanding the global availability of the snow information, we lack streamflow ground data in the areas related to the Eastern part of the Northern Hemisphere. Here, most of the stations with open data are available until 2000^33^, and then the MODIS time frame is not covered. In this way, we were not able to disclose trends and patterns related to this area.

## Results

### Trends in the snow-water nexus

**Fig. 1:**
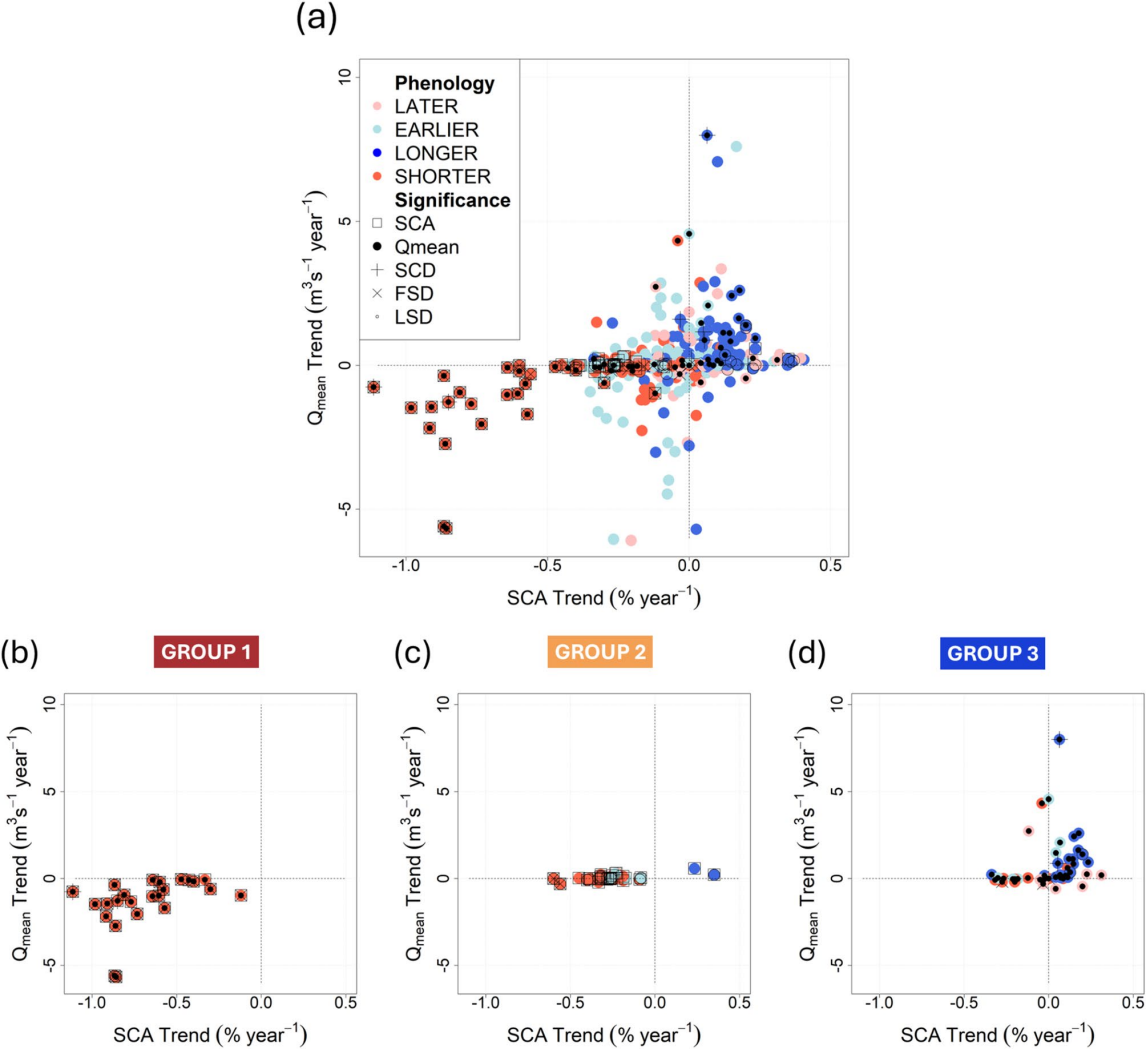
(**a**) Correlation between SCA and Qmean trends over the period 2000–2022 for all catchments (548) and only for those catchments with significant trends: (**b**) in both variables (Group 1, 28 catchments), (**c**) only in SCA (Group 2, 35 catchments), and (**d**) only in Qmean (Group 3, 57 catchments). The colours represent the expected changes in snow season detected by using the snow phenology variables (Fig. 7 in the Methodology section). The symbols (square, point, circle, cross, blade, point) indicate the trends for SCA, Qmean and snow phenology, which are significant at 5% level based on Mann-Kendall statistics (see Method section).

Here, we aim at establishing a relationship between the significant trends in SCA and snow phenological variables (SCD, FSD, LSD) and the mean streamflow values (Qmean) for selected mountain catchments. The correlation between the trends for SCA and Qmean over 548 catchments from the period 2000–2022 is illustrated in Fig. 1.

As expected, (Fig. 1a), we can delineate a general direct pattern relating to SCA and Qmean changes that is the increase/decrease of SCA values reflected in Qmean increase/decrease, which is also evident in the snow cover duration, going from shorter to longer periods. This means that the increase of Qmean is related to a longer snow season (Fig. 1a, dark blue dots), and a decrease in Qmean is related to a shorter snow season (Fig. 1a, red dots). The influence of the other snow phenology variables (FSD, LSD) is taken into account considering the possible variations of the snow season, more specifically a delayed season with delayed LSD (Fig. 1a, pink dots), an anticipated season with an anticipated FSD (Fig. 1a, light blue dots), a longer season with anticipated FSD and delayed LSD (Fig. 1a, dark blue dots), a shorter season with delayed FSD and anticipated LSD(Fig. 1a, red dots) (Fig. 7 in Methods section).

**Fig. 2:**
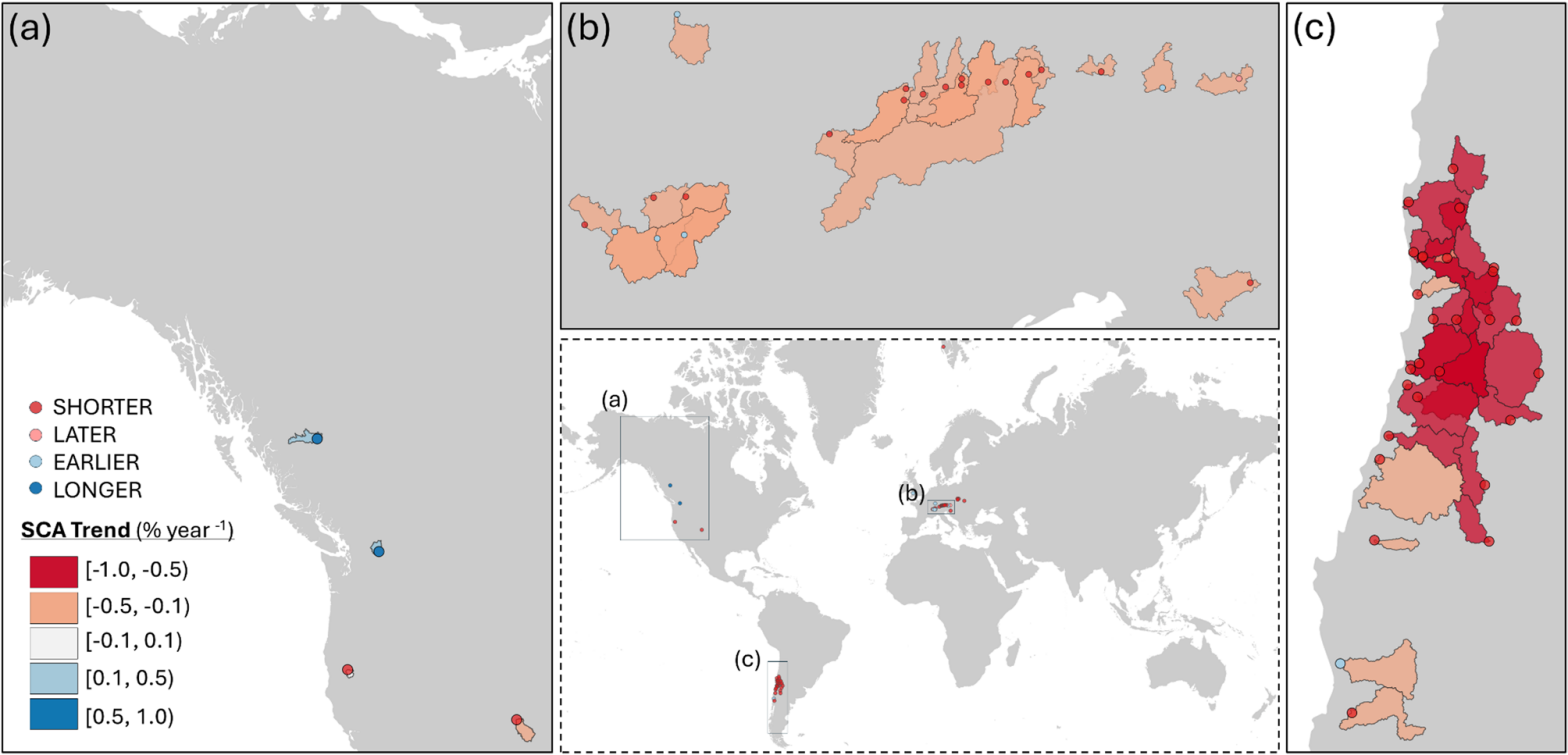
Trends in Snow Cover Area (SCA) in the identified catchments, significant at 5% level based on Mann-Kendall statistics. The dots correspond to the catchment outlet and represent the catchment behaviour regarding the snow phenological behaviour in the snow season (shorter, later, earlier, longer).

**Fig. 3:**
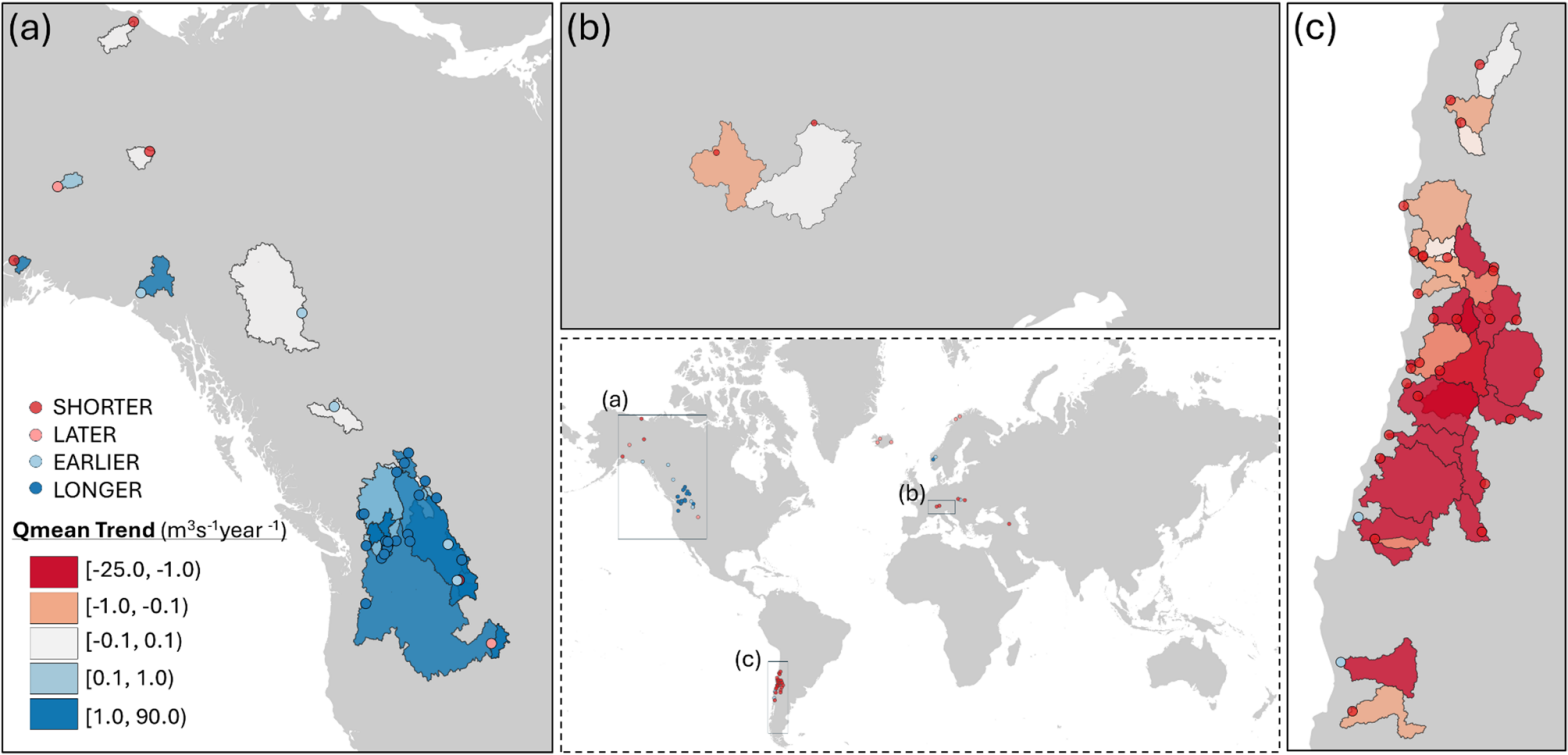
Trends in mean streamflow (Qmean) in the identified basins, at 5% level based on Mann-Kendall statistics. The dots correspond to the catchment outlet and represent the catchment behaviour regarding the snow phenological variables in the snow season (shorter, later, earlier, longer).

Overall, in all the analysed catchments, in only 5% of the cases, the significance of the trends at 5% confidence level coincides in both variables, SCA and Qmean (Fig. 1b), while in 16% of the catchments a significant trend in just one of the variables (Fig. 1c, d) was identified. To geographically locate these catchments, Fig. 2 displays those with statistically significant trends in SCA, while Fig. 3 does so for catchments with Qmean significant trends. In both cases, these trends are represented in relation to the snow phenological variables.

**Fig. 4:**
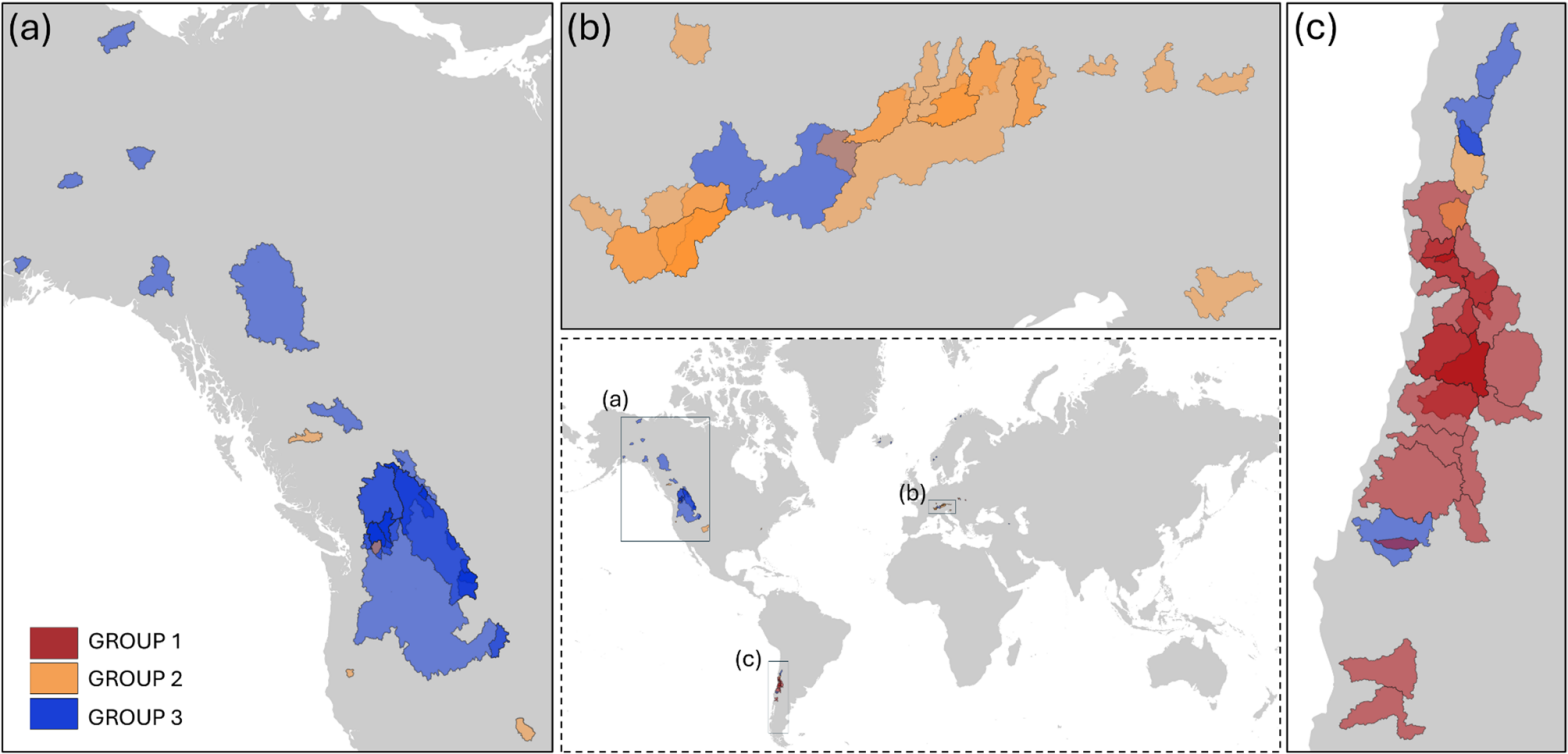
Geographic location of the three groups of catchments. The colours inside the boxes correspond to the changes in the snow seasons, as indicated in Fig. 1.

Therefore, when considering only basins with significant trends, the relationship between SCA and Qmean appears to be clustered into specific groups with distinct behaviours. Three main groups can be identified when considering only catchments with significant changes in SCA or Qmean (Fig. 1, below). Group 1 identifies catchments where both SCA and Qmean report significant changes, while Group 2 represents catchments where SCA changes are significant but the corresponding Qmean changes are not, and finally, Group 3 shows catchments with significant Qmean changes not related to SCA significant variations. To investigate the catchment behaviour further and then relate the observed changes to meteorological patterns, the three identified groups were geographically identified (Fig. 4).

Group 1 comprises 28 catchments, mainly located in the Andes, and only two of them in the Carpathian Mountain range. As mentioned above, this group is characterized by a significant decrease in both SCA and Qmean values. Group 2 has 35 catchments, mainly located in the European Alps. They are characterized by negative trends of SCA values, while the Qmean trends values are close to zero (Fig. 1 below, central panel). Finally, in Group 3 there are 57 catchments, mainly located in North America, where snow season trends indicate an anticipation of the snow start and a delay in snow disappearance, while Qmean values are increasing. Table 1 summarizes the average changes in the snow phenology variables and Qmean for the three identified groups.

**Table 1:** Mean values of the snow phenology (SCA, SCD, FSD, LSD) and Qmean trends and total changes for the three identified groups over the period 2000–2022.

	Qmean (m^3^/s/year)	SCA (%/year)	SCD (days/year)	FSD (days/year)	LSD (days/year)
Trend					
Group 1	− 3.04	− 0.70	− 2.37	0.54	− 1.29
Group 2	0.03	− 0.30	− 0.90	0.20	− 0.48
Group 3	3.20	0.01	0.15	− 0.07	0.01

	Qmean (m^3^/s)	SCA (%)	SCD (days)	FSD (days)	LSD (days)
Total change					
Group 1	− 66.88	− 15.4	− 52.14	11.88	− 28.38
Group 2	0.66	− 6.6	− 19.80	4.40	− 10.56
Group 3	70.4	0.22	3.30	− 1.54	0.15

### Patterns in relation to meteorological drivers, including SWE

**Fig. 5:**
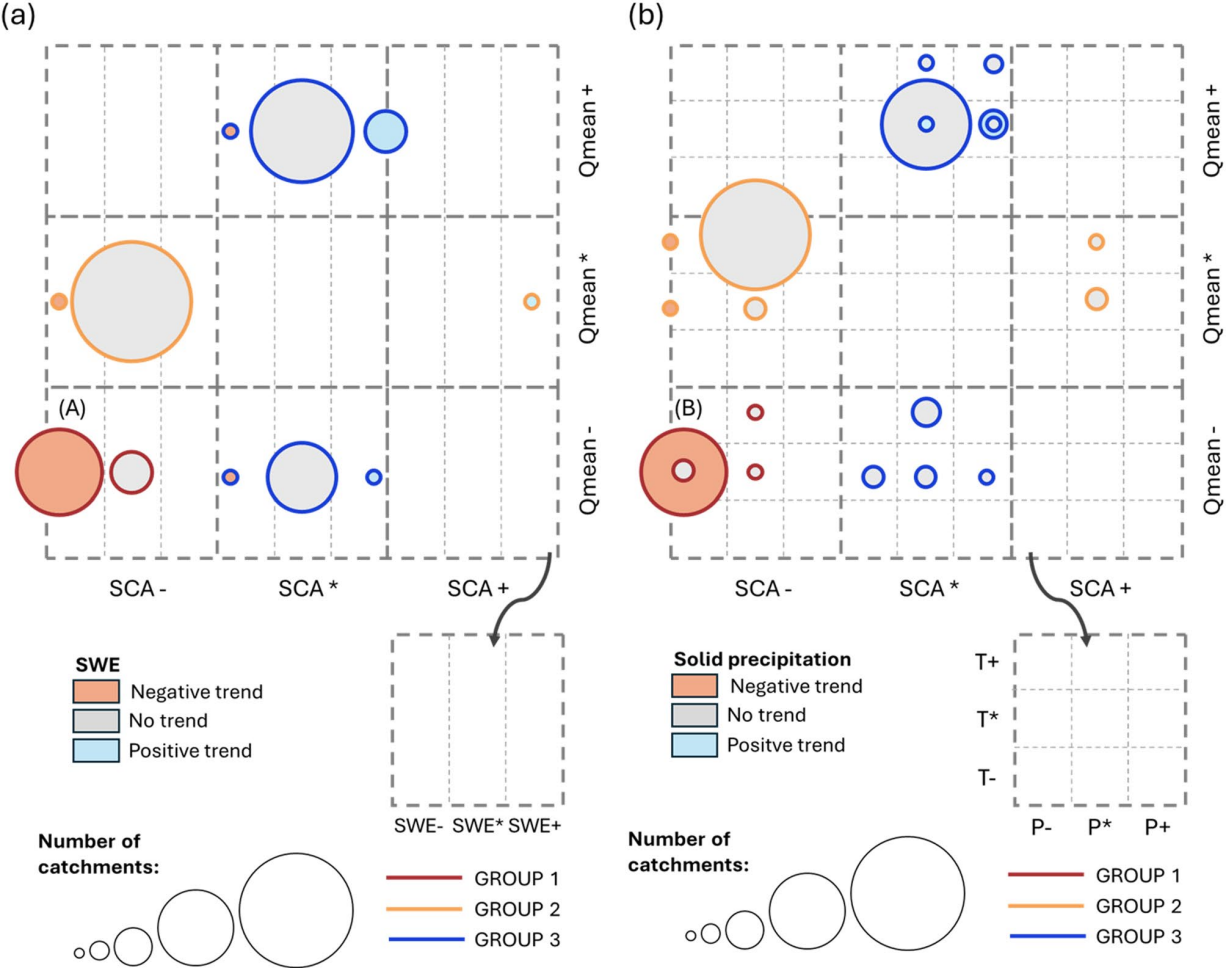
Relationship of the found SCA and Qmean changes in relationship to (**a**) SWE and (**b**) meteorological variables, T, P, S behaviours. The size of the circles indicates the number of catchments where that condition applies. As an example, in case (A), the two circles indicate catchments where decreasing SCA values are associated with a Qmean decrease. The larger circle indicates that in these catchments (n.19), the SCA decrease is also related to SWE decrease, while the smaller circle instead shows the conditions of catchments (n.9) where SWE changes are not significant. Case (B) analyses these catchments in relation to meteorological variables; for most of the catchments (n.21), the location of the circle reports a negligible change in T and a decreasing trend for precipitation, specifically for solid precipitation (the circle is coloured in light red). Only a small group (n.5) (the grey inner circle) has no significant trend in solid precipitation. The other two small circles indicate basins with no trend in T and in P, and no trend in P and a positive trend in T, respectively. These basins belong to Group 1, and are mainly located in South America (Fig. 4). Summarizing, cases (A) and (B) show the conditions of basins with SCA, SWE, and Qmean decrease mainly due to a decrease in solid precipitation.

The observed changes in snow and streamflow variables were correlated with main meteorological factors such as temperature (T), liquid (P), and solid precipitation (S). Moreover, to overcome the limitations of the SCA, which provides only information on snow surface changes, we addressed these SCA changes in relation to SWE values, which represent the variations in the snowpack volume. This final analysis aims to quantify how the surface information provided through the SCA values can be used to identify the main changes in snow mass and then in the Qmean. Figure 5 summarizes the main relationship with SWE and meteorological variables.

#### Group 1

**Fig. 6:**
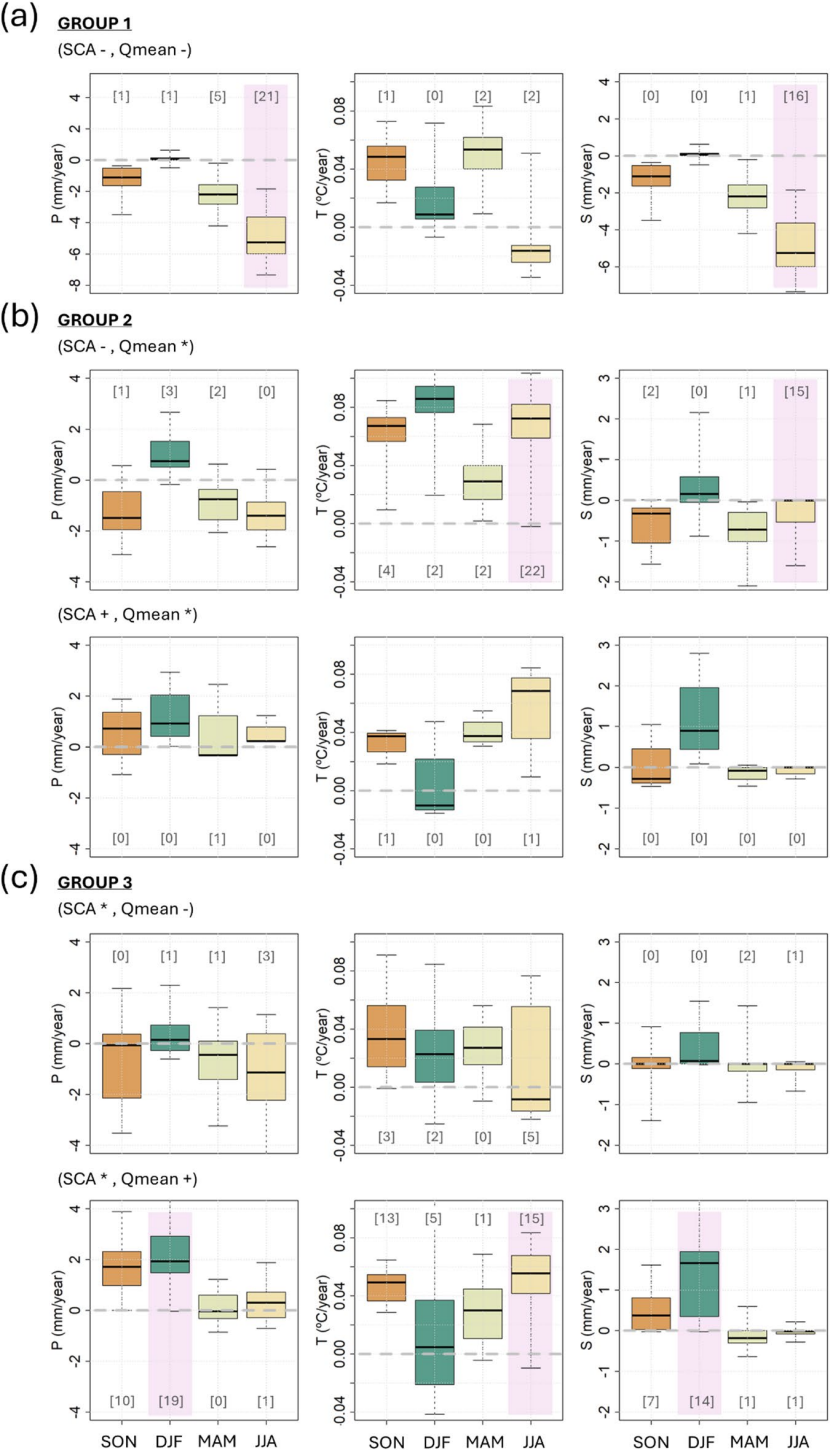
Seasonal trends (SON, DJF, MAM, and JJA) of precipitation (P, left column), temperature (T, central column), and solid precipitation (S, right column) for the catchments of each group. In brackets in each boxplot the number of significant catchments at a 5% confidence level. The purple shadow indicates those groups that show the highest number of catchments (in general higher than 40% of the total number).

For the catchments (n.28) where there is a significant trend in both SCA and Qmean, in all cases, there is a decline corresponding to SWE decrease, mainly due to a decrease in solid precipitation (n.21) (Fig. 5). All but two of these catchments are located in South America (Fig. 4). The remaining two are in the Carpathians. Looking at the different seasons, there is a decrease in snowfall in JJA (winter in the Southern Hemisphere) (Fig. 6a). SWE decreases in both spring and summer (SON, DJF). This is well correlated to anticipated snowmelt (LSD) with a corresponding shorter SCD. In this group, the main driver of SCA and Qmean decrease is the reduction in solid precipitation. Moreover, SCA changes are well related to SWE variations.

#### Group 2

In this group, most of the catchments show a decrease in SCA, but this does not correspond to a decrease in Qmean or SWE (Fig. 5a). Regarding meteorological forcings, the majority of the catchments (80%) are related to a temperature increase, being this increase the main affection to the SCA decrease (Fig. 5b). In some cases, there is a decrease in SCD, but there are no significant changes for FSD and LSD. Considering the different seasons, the primary significant changes in meteorological variables are in summer (JJA) due to temperature increases and decreases in snowfall (Fig. 6b). Nevertheless, the higher temperatures in summer increase melting rates, which can influence the streamflow in the subsequent year. While the streamflow changes in the current year are mainly related to changes in the springtime. This may explain the missing link between SWE, SCA and streamflow. This group is then characterized by temperature increases.

#### Group 3

The catchments are divided into 36 with positive Qmean trends and 17 with negative Qmean trends (Fig. 5a). It is observed that the snow season is generally longer. In approximately 10% of the cases, the increase in Qmean is related to the increase in SWE. When analysing the connection to meteorological variables, for cases where the Qmean decreases, half of them depend on a temperature increase and half on a precipitation decrease (Fig. 5b). When Qmean increases, 30% of the catchments are related to precipitation increases, including an increase in snowfall (Fig. 5b). In these cases, the SCA trends always present a slightly positive value but are not significant at 5% level. There is no precipitation increase for the other catchments, even though the Qmean increases. For this subgroup, a detailed analysis reveals that during the winter months (DJF), an increase in total precipitation is closely associated with a rise in solid precipitation (Fig. 6c). This may lead to higher mean discharge (Qmean) in spring and summer, suggesting that the increase in solid precipitation is likely the primary driver of the observed Qmean rise.

In addition, for a specific catchment, Knik River (Alaska, USA), the increase of Qmean can be related to the presence of glaciers, whose melting can contribute to the Qmean increase.

## Discussion

Notwithstanding the overall snow cover and duration decline observed in mountain regions on a global scale^12,17^, the analysis of the impact on river streamflow indicates a pretty complex behaviour^23^. Indeed, even though the seasonal snow cover represents an undeniable resource for the streamflow in downstream areas, several factors concur to determine its changes.

In South America catchments (Group 1), the decrease of SCA with the consequent reduction in Qmean is mainly due to a decrease in solid precipitation rather than increasing temperature. Since 2010, a sequence of dry years with reduced precipitation from 20% to 40% has been observed^34,35^. Garreaud et al.^34^ confirmed that the reduced precipitation rate in the Chilean mountains determines a reduced snowpack with consequent reduced streamflow. This area is characterized by arid conditions of the basins with a Mediterranean-like climate governed in drought conditions by the El Niño–Southern Oscillation (ENSO). However, the sequence of many years of drought conditions has less than a 5% probability of being due solely to ENSO, and it has been hypothesized to influence anthropogenic climate change, mediated by altered mid- to high-latitude circulation in the Southern Hemisphere^36^. Two of the catchments of this group are located in the Carpathians. Here, a similar behaviour is found with a decreasing trend in SCA and Qmean, which is also reported in the area by Rajwa-Kuligiewicz & Bojarczuk^37^.

For Group 2, mainly located across the European Alps, SCA changes are not associated with corresponding significant Qmean changes. SCA and other snow variables’ declines over this area were addressed and investigated in many studies^6,7,11^. This behaviour is related to a shift from solid to liquid precipitation, more frequent and intense melting resulting from higher air temperatures during winter and spring^6^. Moreover, this warming trend for 50% of the Alpine is associated with the establishment of atmospheric blocking patterns over Europe, even though the correlation between the annual snowpack variability and the NAO is weak and limited to low elevations. Analysis of long-term snow height measurements revealed a decline correlated, especially after 1980, to temperature, precipitation, and atmospheric pattern, thus indicating that the combined effect of these parameters has become more crucial for snowfall frequency^38^. Interestingly, the decline in snow is not reflected in the corresponding streamflow. This aligns with Bard et al.^31^, who found that the annual median flow did not show a significant trend over the whole Alpine region in the last decades.That is, the SCA decline in summer, although reflected in this season, can also affect streamflow in autumn (SON), as a consequence of the composite streamflow regime of the catchments, fed by both glacier and snowmelt^31^. As a result, this contribution is accounted for in the overall streamflow of the following year, diluting the direct nexus between the two variables within the hydrological year.

In the North American catchments (Group 3), mainly located in the northwest of the continent, in general Qmean values with a significant increase are observed, corresponding to a longer snow season. As shown in Fig. 5, this behaviour is also linked to trends in precipitation. This is also confirmed by the study of Liu et al.^39^ across the Northern Hemisphere catchments, even though their results indicate a transition from precipitation to temperature dependency for SWE and streamflow over a longer period from 1950 to 2020. While several studies reported an overall snow decline in the Northern American region, in reality negative patterns are intermixed with positive ones^11,40^. This is also reflected in the trends of streamflow, with variable behaviour. Whitfield & Pomeroy^41^ also report these mixed trends in streamflow over the catchments in this region, whose magnitude and timing are related to regional climate and basin storage. Atmospheric rivers are one of the causes of this variability. They are the leading cause of the increasing winter precipitation in northwestern North America^42,43^ that conditions the increase in streamflow.

These analyses highlight the complex interactions among snow dynamics, hydrology, and meteorological conditions. The timing of temperature and precipitation changes throughout the year plays a critical role in shaping downstream water flow impacts. As noted in the IPCC report on High Mountain Areas^44^, snow-dominated and glacier-fed river basins are projected to experience further changes in both runoff volume and seasonal distribution due to declining snow cover and glacier mass. Additionally, climate model simulations under warming scenarios reveal a substantial reduction in the proportion of meltwater generated at high snowmelt rates. This is primarily attributed to a shortened snowmelt season, which now coincides with periods of lower available energy^19^. Furthermore, the snowpack exhibits a strongly nonlinear sensitivity to temperature, becoming increasingly responsive to each additional degree of warming once winter temperatures exceed − 8 °C. This behaviour helps explain the limited snow loss observed in some regions to date, while also indicating the potential for much sharper declines and increased water security risks in the most densely populated basins under future warming^4^.

These changes have a key role when considering the sectors which are strongly related to water availability such as agriculture and hydropower production^2,6^, thus showing the need for specific adaptation measures. Moreover, the impact of snow changes on the vegetation phenology and greenness is relevant and it was shown that it can surpass the effect of increasing temperature^45^.

This importance of the study is related to data availability. Indeed, for a quantification of the snow and water patterns, consistent and updated data sets are necessary. In this direction, we envisage the further availability of open databases on a global scale and new initiatives to keep the existing databases alive and expand them in regions where the data presence is actually scarce.

## Methods

### Data

This study used three different datasets—snow, streamflow, and meteorological data—during the study period 2000–2022 to derive various variables over the global mountain ranges as delineated by the reference layer Global Mountain Biodiversity Assessment v1.1^46^.

#### Snow

The main snow cover variables addressed in this study are yearly averaged. We have chosen: Snow Cover Area (SCA), Snow Cover Duration (SCD), First Snow Day (FSD), and Last Snow Day (LSD). These variables have been derived using the products NASA-Moderate Resolution Imaging Spectroradiometer (MODIS) Daily L3 Global 500 m Grid, Version 6 (MOD10A1.006). The data are publicly available at https://zenodo.org/records/11181638^47^.

#### Streamflow

This study uses openly available streamflow data from the Global Runoff Database (GRDC). We selected 616 streamflow gauging stations in mountain areas following the GMBA shape file v1.1^46^, which had at least 18 years of data within the study period. Quality checks of these time series were carried out using the criteria proposed by Crochemore et al.^33^. The location of each of these gauging stations defines the outlet of a catchment. When available, the catchment delineation is taken from GRDC’s metadata; when not, they were delineated using the World Hydrological Input Set-up Tool (WHIST; https://hypeweb.smhi.se/model-water/hype-tools/) software, which has been previously used in hydrological studies at global scale^48,49^. When catchments were delineated, the mean SCA for the study period was computed, and only those catchments with an SCA greater than 10%^39^ were selected in the study. Following this criterion, the number of catchments finally considered in the study was reduced to 548.

#### Reanalysis

ERA5-Land monthly averaged data from 1950^50,51^ to the present dataset are used for computing meteorological and snow-derived variables.

### Variables definition

#### Snow variables

To derive the snow variables, the MODIS time series were processed using the online platform Google Earth Engine (GEE), as detailed by Notarnicola^11,47^. The variables are obtained for each hydrological year from 1 October to 30 September of the following year for the Northern Hemisphere and from 1 April to 31 March for the Southern Hemisphere. The data are produced yearly from 2000 to 2022 with a ground resolution of 500 m.

**Fig. 7:**
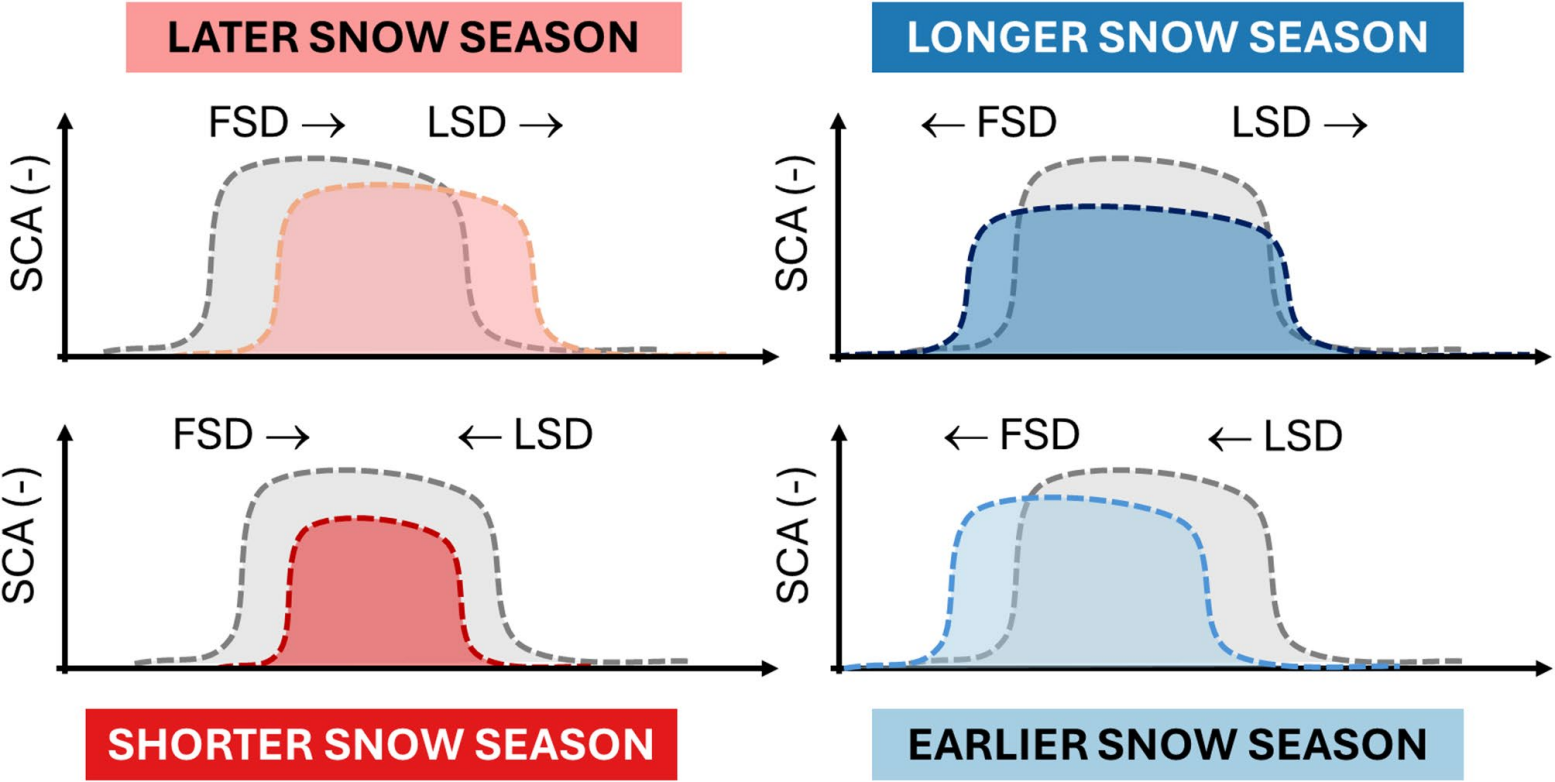
Changes in the snow season related to the changes of the first snow day (FSD) and last snow day (LSD) variables.

In the MODIS product, the Normalized Difference Snow Index (NDSI) was processed to derive the snow cover fraction using the linear approach proposed by Salomonson & Appel^52^. From the snow cover fraction values, SCA values were obtained by averaging over the whole hydrological year. SCD values were derived by using the procedure described in Notarnicola^11^. MODIS SCA products were extensively validated in different mountain areas in the last two decades, indicating an average accuracy of around 94.2%^53^. In mountainous areas, the accuracy was found to be mainly dependent on the illumination conditions and cloudiness. For SCD validation, the comparison with ground data determined the following performances: Mean Absolute Error = 21.1 days, bias = -3.1 days, *R* = 0.84. Readers can refer to Notarnicola^11^ for full details on the snow parameter validation. The SCD, FSD, and LSD trends can determine different behaviours of the snow season as illustrated in Fig. 7. More specifically, a later snow season is determined by a late start of the season (FSD) and a delayed one (LSD). A longer snow season is determined by anticipation of the snow season (FSD) and a later snowmelt (LSD). A shorter snow season is characterized by a later start and anticipated melt. In contrast, an earlier snow season is related to anticipation of the season’s start and end.

#### Streamflow variables

The annual mean daily streamflow (Qmean) for each hydrological year was computed as the variable to characterize the average volume condition of the catchment streamflow. Since our analysis is carried out on an annual basis, Qmean is the variable that best represents this behaviour^54^.

#### Reanalysis variables

Four variables were used. Three were purely meteorological: temperature (T), precipitation (P), and solid precipitation (S). The fourth one was linked to the snowpack characteristics, specifically the snow water equivalent (SWE). The assessment of the datasets is presented in^51^. The quality of meteorological variables is directly linked to the quality of ERA5 and the downscaling algorithm applied to increase the spatial resolution. Precipitation locations and patterns are correctly captured globally, except for the tropical regions^55^. Errors in temperature are smaller than in other reanalysis datasets thanks to the adjustment for the altitude differences using a daily environmental lapse rate^51^. For SWE, there is a general negative bias due to the smoothing of the orography at the resolution of the reanalysis^51^.

No bias adjustment was applied to any of the four ERA5-Land variables used in the study. The possible biases highlighted before are mitigated by accounting for changes rather than actual values. These reanalyses-gridded information was aggregated at the catchment scale by calculating spatial average values for each catchment and temporal aggregation scale.

### Analysis

All previously defined variables were spatially averaged for each of the selected catchments. For SCA, T, and SWE, the mean was used for spatial aggregation, while for P and S, the summation was selected. All these variables were computed at two different temporal scales: annual and seasonal (SON, DJF, MAM, and JJA). In the case of SCD, FSC, LSD, and Qmean, only an annual value was calculated.

The non-parametric Mann-Kendall test^56^ was employed to capture the presence of a time trend in each of the previously defined variables since it is reliable, especially in the presence of outliers with respect to Pearson correlation. In addition, the formulation of Mann-Kendall is suitable for monotonic trends, where there is no seasonal component. The presence of a significant trend is verified by using a Z value. Then, to test increasing or decreasing trends, a two-tailed test is used at α level of significance, where the null hypothesis H0 is rejected if the absolute value of Z is more significant than Z1-α/2. Then, the linear regression’s slope (change per unit time) is estimated using a simple non-parametric procedure developed by Sen^57^. The Sen’s slope uses a linear model to estimate the slope of the trend. The total change during the observed period was obtained by multiplying the slope by the number of years^14^.

## Data Availability

This study used only open available data. Three sources area were used: (1) Snow remotely sensed information derived from MODIS, which is publicly available at [https://zenodo.org/records/11181638] (accessed 2025-04-25); (2) Streamflow data from the Global Runoff Data Centre that can be accessed and downloaded from [https://portal.grdc.bafg.de/applications/public.html?publicuser=PublicUser#dataDownload/Stations] (accessed 2025-04-25); and, (3) ERA5 monthly averaged reanalysis data from the Climate Data Store, which can be freely downloaded from [https://cds.climate.copernicus.eu/datasets/derived-era5-single-levels-daily-statistics?tab=download] (accessed 2025-04-25).
